# An improved X-means and isolation forest based methodology for network traffic anomaly detection

**DOI:** 10.1371/journal.pone.0263423

**Published:** 2022-01-31

**Authors:** Yifan Feng, Weihong Cai, Haoyu Yue, Jianlong Xu, Yan Lin, Jiaxin Chen, Zijun Hu

**Affiliations:** 1 College of Engineering, Shantou University, ShanTou, Guangdong, China; 2 Sangfor Technologies Incorporation, ShenZhen, Guangdong, China; Southwest Jiaotong University, CHINA

## Abstract

Anomaly detection in network traffic is becoming a challenging task due to the complexity of large-scale networks and the proliferation of various social network applications. In the actual industrial environment, only recently obtained unlabelled data can be used as the training set. The accuracy of the abnormal ratio in the training set as prior knowledge has a great influence on the performance of the commonly used unsupervised algorithms. In this study, an anomaly detection algorithm based on X-means and iForest is proposed, named X-iForest, which clusters the standard Euclidean distance between the abnormal points and the normal cluster centre to achieve secondary filtering by using X-means. We compared X-iForest with seven mainstream unsupervised algorithms in terms of the AUC and anomaly detection rates. A large number of experiments showed that X-iForest has notable advantages over other algorithms and can be well applied to anomaly detection of large-scale network traffic data.

## Introduction

In recent years, the network environment has become increasingly complex. Traffic data have exploded and mass infrastructure based on internet of things (IoT) technology and complex networks has had a significant impact on society and the economy [1–3]. Due to the increase in Internet services, network abnormalities including malicious attacks and poor quality of network services have become more diversified and substantially affecting the operation of web services and causing social and economic losses [4–7].

An abnormality is a pattern of data that does not conform to a clearly defined normal behaviour [8]. The cause of the abnormality may be equipment failure or malicious intrusion. To date, anomaly detection as a theme has already been applied in various surveys, review articles and books [9, 10], including the field of network traffic data anomaly detection [11]. It is very difficult to detect abnormal patterns in network traffic data because the types of services provided by the network and the user connection patterns are different, which means that the network traffic has different characteristics and that the pattern distribution is very irregular [12]. In addition, threshold-based anomaly detection methods are more commonly used in the enterprise. There are other cutting-edge unsupervised anomaly detection algorithms, such as a local outlier factor [13] (LOF) and the histogram-based outlier score [14] (HBOS). However, there are currently few methods that can be stable and efficient in the application scenarios of network traffic detection. Therefore, an anomaly detection algorithm that can be applied to high-dimensional large-scale unlabelled data and maintain robustness and high accuracy in a complex and changing network environment is urgently needed.

Isolation forest (iForest) currently have many applications in industry. For example, in the field of semiconductor manufacturing, the high-dimensional and massive characteristics of optical emission spectroscopy (OES) data limit the achievable performance of anomaly detection systems. Puggini and McLoone [15]presented dimensionality-reducing variable selection and iForest-based anomaly detection to solve this problem. The iForest-based method has also been used in studies to detect abnormal situations in the etching process in semiconductor manufacturing and in smart grids, and the effectiveness of the algorithm has been proven through actual industrial data [16, 17]. This paper selects the iForest algorithm [18], which is efficient for massive data, as a basis. iForest can maintain relatively stable detection accuracy in massive data, and its advantages are a short training time and fast detection speed, making it suitable for anomaly detection in many scenarios with massive amounts of data.

However, iForest determines whether a sample is an outlier by calculating the anomaly score of the sample data. The anomaly ratio largely influences the calculation of the anomaly score, so iForest relies heavily on the setting of the anomaly ratio. In the actual industrial network environment, we can rely only on manual experience to set this ratio [19], which means that there is no guarantee that the iForest can achieve the best performance. Inaccurate parameter settings can also lead to low accuracy and high false detection rates in iForest. This paper proposes a solution to this problem, with the following key contributions:

- We propose a new X-iForest method based on iForest, using standard Euclidean distances and upper quartile method to quantify anomaly data.
- We combine X-means [20] with iForest to cluster the standard Euclidean distance values from the data to the cluster centre and effectively classify the data by distance.
- We use a multi-dimensional Gaussian distribution to simulate real network traffic data. Extensive experiments are conducted on 4 simulated datasets and 4 real-world datasets, and the results are compared with those of 6 other cutting-edge unsupervised algorithms.
- Our experimental results show that the proposed X-iForest has significant advantages in the area under the ROC curve (*AUC*) and anomaly detection rate (*ADR*) without the need for an accurate anomaly ratio as prior knowledge.

X-iForest maintains the efficiency of the iForest algorithm in high-dimensional big data training and detection processes while greatly improving the AUC and ADR. When presented with large unsupervised data with no accurate anomaly ratio and large changes in data distribution, the high performance, high accuracy and robustness demonstrated by X-iForest show that the algorithm is suitable for real and complex network traffic anomaly detection scenarios.

This paper is organized as follows: The second part reviews several network traffic anomaly detection methods commonly used in the industrial field. The third part explains the theoretical framework and architecture design of our method. The fourth part evaluates and compares the performance of our proposed method with other methods in the anomaly detection. The fifth part presents our summary and future prospects.

## Related work

Following extensive investigations of actual industrial applications and recently published articles in the field of network health analysis and network traffic anomaly detection, the main methods can be classified as follows.

### Threshold-based approaches

At present, the most widely used methods for network health analysis in the industry are based on active detection, such as ping detection and http detection. On the basis of active detection, a fixed parameter is set based on the experience of operation and maintenance personnel, such as determining a threshold according to a reasonable range of server delay times and then detecting abnormalities in network traffic data based on the threshold. This detection method does not incur excessive computational cost but cannot be flexibly applied to varying network traffic scenarios, especially for the accurate detection of outliers close to the normal distribution, which are difficult to identify accurately and which we define as swing points.

### Statistical-based approaches

Anomaly detection algorithms based on statistics and probability models mainly make assumptions about the distribution of data and determine the “anomalies” defined under the assumptions, so extreme value analysis or hypothesis testing is often used. For example, they might assume a Gaussian distribution for the simplest one-dimensional data and then consider the data whose distance lies outside a specific range as anomalous points. After generalizing to high dimensions, one can assume that each dimension is independent and add the abnormality of each dimension. The most representative algorithm is HBOS. Paulauskas and Baskys [21] used the HBOS to detect anomalies in a computer network concluded that the HBOS with dynamic bins showed better results than other approaches in detecting rare events. The HBOS algorithm, which is based on the assumption of independence of each dimension of multi-dimensional data, divides *n* partitions in each dimension. The outlier corresponding to each interval depends on the density. The higher the density is, the lower the value. If the statistical assumptions are true, then this method is simple and very effective. However, this type of statistical method relies heavily on the distribution assumption of the data set, which also causes the robustness of this type of algorithm in the real data set to be relatively poor.

### Distance-based approaches

The distance-based method is generally regarded as the basic method of outlier detection research. The distribution of abnormal points is different from that of normal points, so a series of algorithms are derived to identify abnormal points through distance-representing similarity. Wang et al. [22] proposed a distance-based proximity detection method using the K-nearest neighbour (KNN) algorithm to analyse and detect the abnormal flow data in a wireless sensor network (WSN). The KNN classifier is used for anomaly detection. For parameters *k* and *n*, the k-nearest neighbour distance is calculated for each point. The top *n* points are taken as anomalies by sorting the k-nearest neighbour distances in descending order. They implemented and tested the QualNet simulation platform and proved the effectiveness of the proposed KNN algorithm through simulation results. The principal component analysis (PCA)-based anomaly detection algorithm can also be regarded as a distance-based method. One method is to find k feature vectors and then calculate the weighted Euclidean distance from each sample to the hyperspace formed by these feature vectors and use it as the sample abnormality. Hoang and Nguyen [23] investigated the application scenarios of PCA algorithm and proposed a new general formula for distance calculation and a PCA-based IoT detection method. They verified the feasibility of their proposed method through a variety of experiments. Distance-based approaches incur a very high computational cost for massive datasets, with loss of performance when applied to network traffic anomaly detection.

### Density-based approaches

These approaches introduce the concept of LOF, in which each instance is assigned a score based on the neighbours’ local density denoting a degree of outlierness. A potential outlier is identified by the relatively high LOF value. Based on this main idea, some extended models have been proposed. Gan and Zhou [24] combined the LOF with the Density-based spatial clustering of applications with noise (DBSCAN) algorithm to realize the adaptive dynamic adjustment of parameters to changing data. The method was used to optimise the accuracy of network traffic scenarios. The experimental results show that the method based on the improved LOF algorithm has high practical application value. The cluster-based local outliers factor (CBLOF) algorithm uses K-means to pre-classify the data before performing the LOF algorithm [25] and then uses the LOF algorithm separately for the data in each cluster. The problem with this type of method is the same as that of the distance-based method: a considerable amount of calculation when facing large, high-dimensional data. Moreover, density-based anomaly detection algorithms require relatively extensive prior knowledge and experience in the selection of parameters, which also limits the application of such algorithms to fast-changing network traffic anomaly detection.

### Neural network-based approaches

In network traffic anomaly detection tasks, network traffic datasets are often massive and complex. In recent years, neural network-related algorithms have been proven to be well applied in complex scenarios and have very good performance [26]. Kim and Cho [27] proposed the C-LSTM method to extract more complex features by combining a coevolutionary neural network (CNN), long short-term memory (LSTM) and deep neural network (DNN) and verified in experiments that its performance is better than that of other state-of-the-art machine learning techniques. Wei and Wang [28] combined a convolutional neural network (CNN) and recurrent neural network (RNN), proposed a network anomaly detection method based on hierarchical spatiotemporal feature learning (HAST-NAD), and proved that the time series characteristics algorithm performs better than the spatial characteristics algorithm. Although deep learning has shown excellent performance in many tasks, it relies heavily on high-quality labelled data. In the actual industrial scenario of network traffic anomaly detection, it is often difficult to obtain a large amount of labelled data, which means that the use of neural network-based methods face the problem of cold starting. Moreover, network traffic data have different distributions on different servers, different applications, and different times; thus, generalization is a severe problem faced by neural-network-based methods.

### Isolation forest-based approaches

Since the data in network health analysis and network traffic anomaly detection scenarios often involve large data volume, high-dimensional data and a small proportion of anomalies, iForest are very suitable for network traffic anomaly detection. Compared with other algorithms, iForest can maintain higher detection performance and accuracy with massive data. IForest is used as an ensemble learning method, which contains multiple isolation trees. Each tree can be regarded as a weak classifier. The training set used for each weak classifier is obtained by random sampling from the full dataset using the bootstrap method. The final result is obtained by combining the results of all the isolation trees, which means that the iForest has a good generalization performance and can largely prevent overfitting. Hariri et al. proposed an extended isolated forest algorithm(EiForest) [29] that allows the branching hyperplanes to take on any slope as opposed to hyperplanes only parallel to the coordinate frame. EiForest addresses the impact of bias introduced in the standard isolated forest case on the anomalous scores for a given data point. The experimental results show that the EiForest possesses stronger robustness. Although the EiForest solves the problem caused by bias, it does not take into account the effect of the abnormal ratio on the detection results. Ding and Fei [30] used a sliding window frame and proposed an adaptive streaming data anomaly detection algorithm iForestASD based on iForest, which can be used to detect network traffic data generated in applications such as computer networks and sensor networks, and used experiments to prove that their proposed algorithm can effectively detect anomalies in the data stream. Puggini and McLoone proposed an anomaly detection method based on reduced dimensional variable selection and isolated forests [15]. The method is used to solve the problem that the high dimensionality and correlation of optical emission spectral data limit the performance of anomaly detection systems, and it is more interpretable compared to methods such as PCA. Wang et al. used the isolated forest algorithm for aero-engine fault detection [31], and the experimental results showed that the method has higher detection accuracy and shorter running time. Alsini et al. combined the local outlier factor (LOF) algorithm with isolated forests to solve the limitations of LOF in evaluating concrete mixtures [32], and experimental results demonstrated that the method was more effective in detecting anomalous sequences. Chen et al. combined Gaussian mixture model (GMM) with isolated forest for anomaly detection and identification of key behavioral attributes in continuous state monitoring (CM) data [33], and experimentally demonstrated that the method is more effective for high-dimensional data compared with other methods. Zhou et al. used the isolated forest algorithm to predict the final welding quality [34], and the experimental results showed that the isolated forest possessed better classification performance. Wang et al. proposed a general separation method based on linear prediction analysis and isolated forest [35], which separate multi-source partial discharge signals and distinguish various partial discharge signals. The experimental results confirmed that the method can effectively separate and distinguish various partial discharge signals. Ahmed et al. use isolated forests to detect covert data integrity assault (CDIA) utilizing non-labeled data in smart grid (SG) communication networks [17], and simulation results show that the method can handle non-labeled historical measurement datasets well and significantly improve attack detection accuracy. Xing et al. used isolated forest to identify the interest flooding attack [36], and the simulation results showed that the method has high attack detection accuracy and fast detection speed.

However, the unavailability of sufficient high-quality data and the requirement for a priori knowledge make these iForest-based methods still difficult to apply to real network traffic monitoring scenarios. We introduce X-Means and standard Euclidean distances to quantify the anomalies of the data for this problem, and address the problem of not having sufficient a priori knowledge and labeled data in realistic anomalous traffic monitoring.

## Materials and methods

This section describes our approach and the algorithmic tools used. The proposed X-iForest is composed of iForest and X-means. After obtaining the normal clustering centres by iForest, the standard Euclidean distance from the data points to the normal clustering centres was used to quantify the degree of abnormality. The degree of abnormality was clustered using X-means after filtering the extreme values by the upper quartile method. Finally the 2-means method was used as a classifier to classify the data into normal and abnormal classes.

### X-means

X-means clustering is an improved algorithm based on K-means [20]. It is used to solve the main problem of K-means clustering, which requires prior knowledge about the number of clusters. In this method, the number of clusters *k* is estimated in an unsupervised manner based on the data set itself, using *k*_max_ and *k*_min_ respectively as upper and lower limits for the possible values of *x*. In the first step of X-means grouping, X-means performs a clustering where *x* = *k*_min_. In the next step, each cluster is regarded as an initial parent category, and a calculation is performed on each parent category which is calculates the *BIC* scores before and after classification and compares them to decide whether to divide the parent class into two groups. The *BIC* score, which helps determine the best representation for sample data, is defined as:

$$\begin{array}{c} BIC\left(\phi \right)=\hat{l_{\phi }}\left(D\right)-\frac{P_{\phi }}{2}\cdot \text{log}\;R \end{array}$$ (1)

where *ϕ* represents the model and $\hat{I_{\phi }}\left(D\right)$ is the log-likelihood of the data according to the *ϕ* model, and taken at the maximum likelihood point. *P*_*ϕ*_ is the number of parameters in model *ϕ*. For example, model *ϕ*_2_ is better than model *ϕ*_1_ if *BIC* (*ϕ*_2_) > *BIC* (*ϕ*_1_).

In this way, clusters provide an accurate distribution of samples. As a result, the algorithm either replaces the parent generation with the centroid or maintains the centroid and keeps it. Then, the structure is continuously updated according to each choice until the estimated number of clusters reaches *x*_max_ or has converged to the best structure.

### Isolation forest algorithm

**Algorithm 1** iTree(*D*, *h*, *l*)

**Input**: *D* = (*x*_1_, *x*_2_, …, *x*_*n*_)—dataset,*h*—the height of tree,*l*—height limit

**Output**: an iTree *t*

**Initialize**: *t* = ⌀

1: **if** *h* >= *l* or *Size*(*D*) <= 1 **then**

2:  **return** *t*

3: **else**

4:  randomly select *q*_*i*_ a feature of *D*

5:  randomly select a split point *p* ∈ (min(*q*_*i*_), max(*q*_*i*_))

6:  *D*_*l*_ ← filter (*D*, *q*_*i*_ < *p*)

7:  *D*_*r*_ ← filter (*D*, *q*_*i*_ >= *p*)

8:  repeate iTree(*D*_*l*_, *h* + 1, *l*) and link the obtained tree as the left tree of *t*

9:  repeate iTree(*D*_*r*_, *h* + 1, *l*) and link the obtained tree as the right tree of *t*

10: **end if**

The iForest was proposed by Liu et al. [18]. It has many applications in the industrial field and involves an ensemble of isolation trees, similar to random forests and decision trees. By definition, an iForest is composed of a certain number of isolation trees:

$$\begin{array}{c} IF=\{t_{1},\ldots ,t_{T}\} \end{array}$$ (2)

where iTree is obtained according to the flow of Algorithm1. The path length *h*(*x*) of the sample *x* is the number of edges passed from the root node of the isolation tree to the leaf node. The average number of steps required to isolate a sample *x* in a forest is then

$$\begin{array}{c} h\left(x\right)=\frac{1}{T}\sum_{t\in IF}h_{t}\left(x\right) \end{array}$$ (3)

Intuitively, clusters with high density need to be cut many times to be isolated, but those with low density can easily be isolated. Under this random segmentation strategy, abnormal points usually have shorter paths. Let *n* be the number of samples in the data set, *c*(*n*) be the average path length of the tree, and *c*(*n*) be defined as:

$$\begin{array}{c} c\left(n\right)=2H\left(n-1\right)-\frac{2(n-1)}{2n} \end{array}$$ (4)

where *H*(*i*) is the harmonic number estimated as:

$$\begin{array}{c} H(i)\approx \text{ln}\;(i)+0.5772156649 \end{array}$$ (5)

The normalized anomaly score *s*(*x*, *n*) of sample is defined as:

$$\begin{array}{c} s\left(x,n\right)=2^{\frac{h(x)}{c(n)}} \end{array}$$ (6)

If instances return *s* very close to 1, then they are definitely anomalies, if instances have s much smaller than 0.5, then they are quite safe to be regarded as normal instances, and if all the instances return *s* ≈ 0.5, then the entire sample does not really have any distinct anomaly.

### X-iForest: Improved isolation forest based on X-means

Although iForest are more suitable for massive unlabelled data than other algorithms to a certain extent, similar to other unsupervised algorithms, the performance of the algorithm is very dependent on the settings of the abnormal ratio. The actual network conditions are very complicated, causing the definition and data distribution to change very quickly, so it is necessary to always use the newly captured unlabelled data as the training set to update the model. In this case, we can rely only on manual experience to determine the value of the abnormal ratio, but an inaccurate abnormal ratio would destabilize the performance of the iForest, resulting in low accuracy and a high false detection rate. In this study, the X-means clustering algorithm is used to improve the iForest to improve the performance of algorithm anomaly detection when the real anomaly ratio is unknown, even exceeding the performance of the original iForest algorithm with an accurate abnormal ratio.

According to the actual situation in industry, we treat the dataset as a completely unlabelled dataset; that is, the training set has no labels, and the abnormal ratio is unknown. Our algorithm is described as follows:

**Algorithm 2** X-iForest Algorithm

**Input**: *D* = (*x*_1_, *x*_2_, …, *x*_*n*_)—dataset, *k*_*max*_—the upper limit of the number of clusters in X-means

**Output**: the index list of normal values *Nlist*, the index list of abnormal values *Alist*

**Initialize**: iForest,*Nlist* = ⌀,*Alist* = ⌀, distance list *Dlist* = ⌀

1: set the contamination parameter of iForest to 2 times the background knowledge of the relevant field

2: get preliminary classification result *L* = (*l*_1_, *l*_2_, …, *l*_*n*_) of *D* with iForest

3: **for** each label *l*_*i*_ in *L* **do**

4:  **if** *l*_*i*_ = 1 **then**

5:   *Nlis* append *x*_*i*_

6:  **else**

7:   *Alist* append *x*_*i*_

8:  **end if**

9: **end for**

10: calculate the cluster center *C* of *Nlist*

11: **for** each sample *s*_*i*_ in *Alist* **do**

12:  calculate the standard Euclidean distance *d*_*i*_ between *s*_*i*_ and *C*

13:  *Dlist* append *d*_*i*_

14: **end for**

15: *MAX* ← box plot (*Dlist*)

16: *Dlist* ← filter (*Dlist*, *d*_*i*_ < *MAX*)

17: perform X-means clustering on *Dlist* and calculate the cluster center of each cluster to get the result *X* = {*xclu*_1_, *xclu*_2_, …, *xclu*_*k*_}, where *k* <= *k*_*max*_

18: perform K-means clustering with *k* = 2 on *X* and get the result *K* = (*kclu*_1_, *kclu*_2_), where the center of *kclu*_1_ < *kclu*_2_

19: **for** each sample *s*_*i*_ in *kclu*_1_ **do**

20:  *Nlist* append *s*_*i*_

21:  *Alist* remove *s*_*i*_

22: **end for**

23: **return** *Nlist*,*Alist*

For other algorithms that require a priori knowledge, they need to learn the true abnormal ratios of many datasets to do a priori fitting. The closer the regression results are to the true ratios the more effective these algorithms will be, and once they encounter a little noise (which is extremely common and unavoidable in reality) the algorithms will be much less effective or even ineffective. Such algorithms, which rely on a priori knowledge, are not stable in reality. Our proposed algorithms can perform well as long as they have basic background knowledge of the scenario in a real-world application. For example, in network traffic anomaly detection, the usual anomaly rate in this domain is about 0.02 to 0.05, and we simply need to set the parameters higher than that to achieve excellent detection results. In contrast, we prefer to call our algorithm background knowledge-based rather than a priori knowledge-based.

First, iForest are used for preliminary detection, and the contamination parameter is seted to 2 times the background knowledge of the relevant field. This is to detect as many outliers as possible. In actual situations, the cost of misclassifying an abnormal value as a normal value is higher than the misevaluation of a normal value as an outlier. The first task should be to detect as many outliers as possible. Since our estimated anomaly ratio is higher than the true ratio, there may be cases where the normal value is misclassified as an outlier after the initial detection. We need to minimize misclassification as much as possible while maximising the abnormal detection rate. This part is reflected in lines 1 to 8 in Algorithm 2.

Second, we calculate the cluster centre of this part of the normal data in the preliminary detection and then calculate the standard Euclidean distance from the suspected abnormal value to the normal cluster centre. The standardized Euclidean distance is an improvement scheme intended to address the shortcomings of the simple Euclidean distance [37], mainly for data with large differences in data distribution in each dimension.

Let ***a*** = (*x*_11_, *x*_12_, …, *x*_1*n*_) and ***b*** = (*x*_21_, *x*_22_, …, *x*_2*n*_) be the observed data, where *s*_*n*_ is the standard deviation and where the standardized Euclidean distance between ***a*** and ***b*** is:

$$\begin{array}{c} d=\sqrt{\sum_{k=1}^{n}(\frac{x_{1k}-x_{2k}}{s_{k}}{)}^{2}} \end{array}$$ (7)

The abnormal ratio assumed in the preliminary calculation is higher than the true ratio, so the data evaluated to be normal after the initial detection are the data whose distribution in the data set most conform to the normal definition and do not include swing points. The closer the distance to the cluster centre is, the closer the data are to the normal data distribution, and the farther the distance is, the higher the abnormality of the data. This step is shown in lines 9 to 13 in Algorithm 2.

Third, extreme values in the distance value are filtered out through box plot [38]. Let *Q*_1_ be the upper quartile and *Q*_3_ be the lower quartile. The *MAX* and *MIN* observations can be defined as:

$$\begin{array}{c} MAX=Q_{1}-1.5\times (Q_{3}-Q_{1}) \end{array}$$ (8)

$$\begin{array}{c} MIN=Q_{3}+1.5\times (Q_{3}-Q_{1}) \end{array}$$ (9)

Extreme values affect subsequent clustering by causing the overall characteristics of the data to shift. Therefore, in this step, we filter out extreme values whose distance value is greater than the *MAX* observation value, and classify these points directly as anomaly values. Since the Euclidean distance calculation result is not less than 0, we do not consider the case of values less than the *MIN* observation value. This part is reflected in line 14 and line 15 in Algorithm 2.

Fourth, the distance value calculated in the second step is used for X-means clustering with *k*_*min*_ = 2. This setting is used because this part of the data contains misdetected normal values and real abnormal values, so these data should be divided into at least 2 clusters to prevent the data distribution from being too uniform and causing X-means to collect the data into 1 cluster. Line 16 in Algorithm 2 are this part.

Finally, we calculate the standard Euclidean distance from the cluster centre of each cluster obtained by X-means clustering to the normal cluster centre. Then, K-means clustering with *k* = 2 is performed on these distance values, and all clusters are divided into normal and abnormal categories. This step is shown in lines 17 to 22 in Algorithm 2.

**Fig 1:**
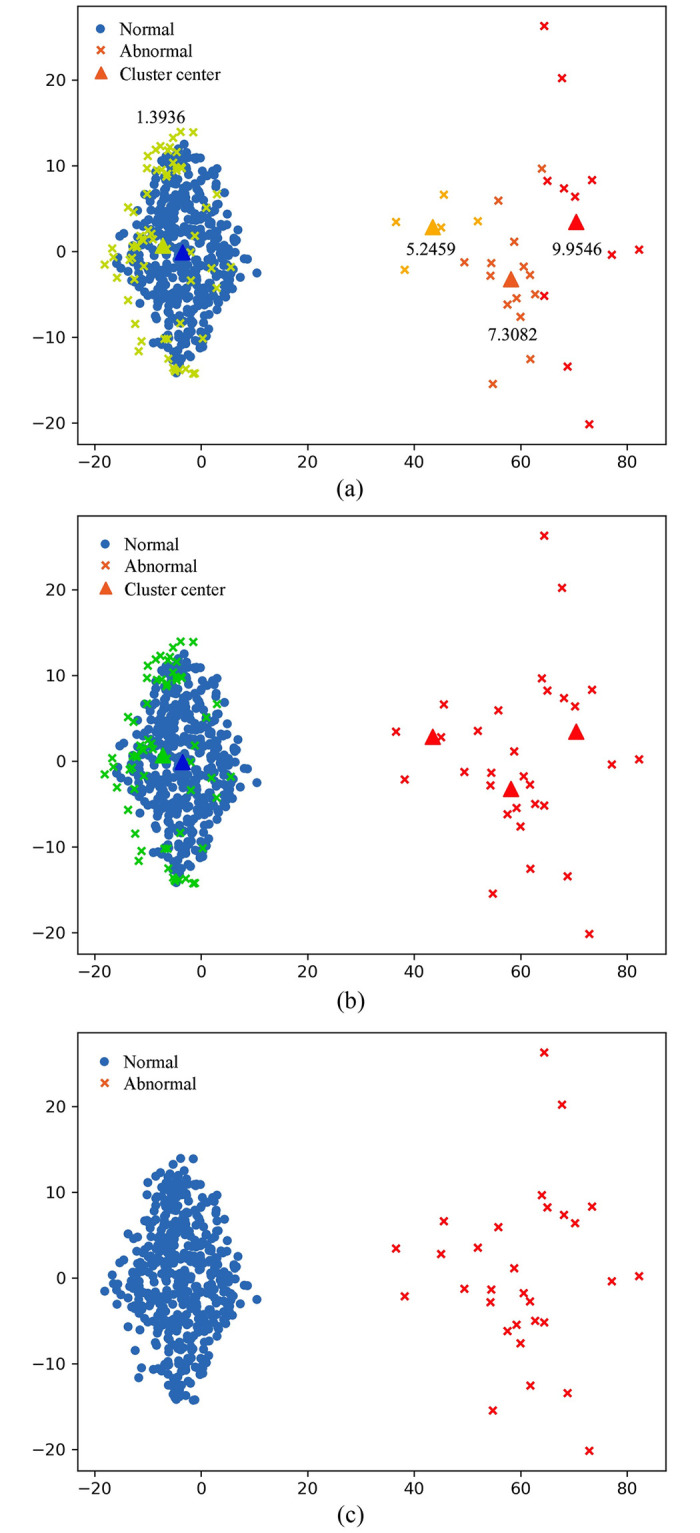
The result of X-means clustering on standard Euclidean distance from the abnormal cluster centers to the normal cluster center. (a): The result of K-Means clustering. (b): The final anomaly detection result of the generated data bu using X-iForest. (c): Demonstration of X-iForest on a test dataset.

To show the process of X-iForest more intuitively, we determine the approximate range of normal and abnormal values based on the distribution of actual network traffic data and randomly generated a simulation data set within the distribution range to demonstrate the steps of the algorithm. We simulated normal data $X=(x_{1},\ldots ,x_{6})\in R^{n\times 6}$ with *n* = 500 samples and the abnormal data $Y=(y_{1},\ldots ,y_{6})\in R^{n\times 6}$ with *n* = 30 samples. For each feature, the elements in *X* and *Y* are independent random samples of normal distribution and abnormal distribution, respectively. The data set has a total of 530 samples, each of which has 6 dimensions. The results after X-means clustering are shown in Fig 1(a). To make the results more intuitive, we use PCA for dimensionality reduction [39]. The triangle represents the centre of a cluster, and the point with the same colour as the triangle represents the data grouped into that category. The blue dot represents the normal data detected in the first step. The marked numbers represent the distance from the centre of each cluster to the centre of the normal cluster. The abnormal data are divided into 4 clusters by X-means. The smaller the standard Euclidean distance from the centre of the normal cluster is, the closer the cluster is to the normal data distribution.

The result of K-means clustering is shown in Fig 1(b); the smaller type is shown in green, which represents that the points contained in the clusters of this type are close to the normal data distribution of the preliminary detection, so these points are considered to be normal values that were misevaluated as abnormal and reclassified into the normal class. The data contained in the other cluster are shown in red, representing classification as anomaly data. Fig 1(c) shows the final anomaly detection result.

## Experiments

### Evaluation metric

The metrics for evaluating outlier detection are the *ADR* and *AUC*. The *ADR* refers to the ratio of detected abnormal values to all abnormal values. In industrial applications, the cost of erroneous anomaly detection is much greater than the cost of false detection of normal values. The first task of an anomaly detection algorithm in practical applications should be to identify outliers as much as possible, so the *ADR* is an important evaluation indicator in industrial applications. *DN* is the number of abnormal data detected correctly, and *AN* is the number of abnormal data in the entire test set. The *ADR* is defined as:

$$\begin{array}{c} ADR=\frac{DN}{AN} \end{array}$$ (10)

The *AUC* is calculated from the recall (*REC*) and false positive rate (*FPR*). Let *TP* be the number of detected true positives, *FP* the number of detected false positives, *TN* the number of detected true negatives and *FN* the number of detected false negatives. The recall and false positive rate are defined as follows:

$$\begin{array}{c} REC=\frac{TP}{TP+FN} \end{array}$$ (11)

$$\begin{array}{c} FPR=\frac{FP}{FP+TN} \end{array}$$ (12)

### Data

To better evaluate the performance of algorithms in different network environments, we used four simulation datasets, which are obtained by simulating the real network traffic data captured on the load balancer. We desensitize the existing real data, expand the real data set according to the distribution of real network traffic data. The simulated data are sampled from a mixture of two multivariate Gaussian distributions. The multivariate Gaussian distribution mixture model can be represented by the following equation [40]:

$$\begin{array}{c} p\left(x;\mu ,\Sigma \right)=\frac{1}{{\left(2\pi \right)}^{\frac{n}{2}}{\left|\Sigma \right|}^{\frac{1}{2}}}\text{exp}(-\frac{1}{2}{\left(x-\mu \right)}^{T}\Sigma^{-1}\left(x-\mu \right)). \end{array}$$ (13)

where *n* is the dimension of the feature and the nondimensional vector *μ*_*i*_ is defined as the mean value of the i-th feature, ***μ*** = (*μ*_1_, *μ*_2_, …, *μ*_*n*_) is a n-dimensional vector. Here, Σ is the covariance of the feature, which is an n-dimensional matrix:

$$\begin{array}{c} \Sigma =[\begin{array}{ccc} \sigma (x_{1},x_{1}) & \cdots & \sigma (x_{1},x_{n})\\ \vdots & \ddots & \vdots \\ \sigma (x_{n},x_{1}) & \cdots & \sigma (x_{n},x_{n}) \end{array}]\in R^{n\times n} \end{array}$$ (14)

The multivariate Gaussian distribution is chosen because it is the maximum entropy distribution for a given mean and variance [40]. Therefore, a minimum number of assumptions are imposed on the simulation data. We usually assume that there is a correlation between the various features of network traffic data and that a multivariate Gaussian distribution can automatically capture the correlation between the features, which makes this approach suitable for the simulation of network traffic data. We use two multivariate Gaussian distributions to simulate the normal data and abnormal data of real network traffic data and sample them to form simulation datasets. The configuration of the 4 simulation datasets is as follows:

**Simulation 1**. The first simulation dataset has a total of 6-dimensional features; the training set contains 1 million samples, and the abnormal ratio is approximately 2%. The dataset is characterized by a large amount of data, and abnormal points are abnormally distributed in all dimensions. According to the above characteristics, the distribution of the abnormal data is quite different from that of the normal data, so the challenge of abnormality detection is less difficult.
**Simulation 2**. The abnormal ratio, number of samples, and dimension of the simulation2 set have the same settings as simulation1. However, each anomaly point presents anomalies in a few random features only. We assume that the features are independent of each other in the simulation2, so the covariance matrix Σ can be expressed as:
  $$\begin{array}{c} \Sigma =[\begin{array}{cccc} \sigma_{1}^{2} & 0 & \cdots & 0\\ 0 & \sigma_{2}^{2} & \cdots & 0\\ \vdots & \vdots & \ddots & \vdots \\ 0 & 0 & \cdots & \sigma_{6}^{2} \end{array}]\in R^{6\times n} \end{array}$$ (15)
  We randomly select 3 features from 6 features, calculate the variance and mean of these 3 features from normal data, and use the variance and mean of abnormal data for the remaining 3 features. After the combination, Σ and *μ* are obtained, and a multi-dimensional Gaussian distribution of abnormal data is constructed. Compared with the first simulation data set, there are more swing points. Swing points arise when the point is at the edge of the normal data distribution and abnormal data distribution, presenting a challenge in the anomaly detection task. Compared with simulation1, the data distribution in simulation2 is more challenging as a task of abnormal data detection.
**Simulation 3**. The third simulation dataset uses the configuration of simulation1 with the same proportion of outliers and number of dimensions. The number of samples in the training set in this data set is only 730 to test the performance of the outlier algorithm with a small sample data set.
**Simulation 4**. The fourth simulation dataset uses the configuration of simulation1 with the same proportion of outliers. The difference is that the dimension of the fourth data set is extended to as high as 50, and the data distribution of a few dimensions is quite different from that of other dimensions. In addition, to simulate the existence of the real data set, some extreme values are randomly added to the abnormal points of the data set. The challenge of this data set to the abnormal detection algorithm lies in the high dimensionality of the data set and the influence of extreme values.

### Other datasets

To make the experimental data more convincing, we measure the performance of our proposed method for detecting outliers through a number of publicly available datasets. The Shuttle, Satellite, and BreastW datasets are selected from the UCI data set, and the Mulcross data set is selected from the ODDS data set [41]. Table 1 lists the statistical characteristics of each data set. These datasets contain different cardinal number, number of attributes and anomaly proportions, which means that they can be used to test the robustness of the algorithms, which is key to their ability to perform well in complex and changing network environments.

**Table 1:** Statistical characteristics of the other experimental dateset.

Dataset	Cardinal number	Number of attributes	Abnormal points
Shuttle	49097	9	3437
Mulcross	262144	4	26214
Satellite	6435	36	2036
BreastW	683	9	239

### Verification of algorithm validity

**Fig 2:**
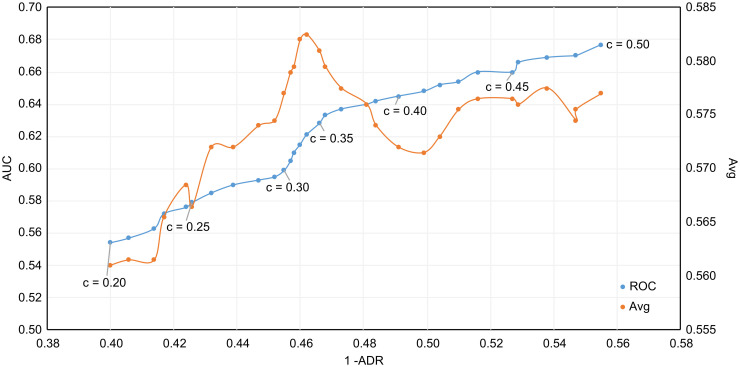
The performance of iForest under different contamination parameters in the dataset with a abnormal ratio of 0.32, c represents the contamination parameter.

We used the *AUC* and *ADR* to jointly evaluate the performance of iForest under different contamination parameters. The satellite dataset from the ODDS dataset is used here, and the abnormal ratio of the training set is 0.32. Fig 2 plots the ROC curve for simultaneous assessment of *AUC* and *ADR* and the Avg curve for the sum average of *AUC* and *ADR*. Near the true abnormal ratio(0.32) of the data set, the point of the ROC curve is closest to the upper left corner, indicating that the point in this area is closest to the optimal critical value. Moreover, the Avg curve also reaches the highest value at *contamination* = 0.34, which is near the true anomaly ratio. This proves that an accurate abnormal ratio has an important impact on the performance of iForest.

To verify the effectiveness of the X-iForest algorithm, we tested EiForest, iForest, LOF, CBLOF, PCA, HBOS, and KNN on a total of 8 datasets mentioned earlier and compare them with X-iForest. For the 8 datasets, we use the same training set and test set for all algorithms, and adjust the abnormal ratio of other algorithms except X-iForest to the true abnormal ratio to optimize the performance of these algorithms and ensure the objectivity of the experiment.

**Fig 3:**
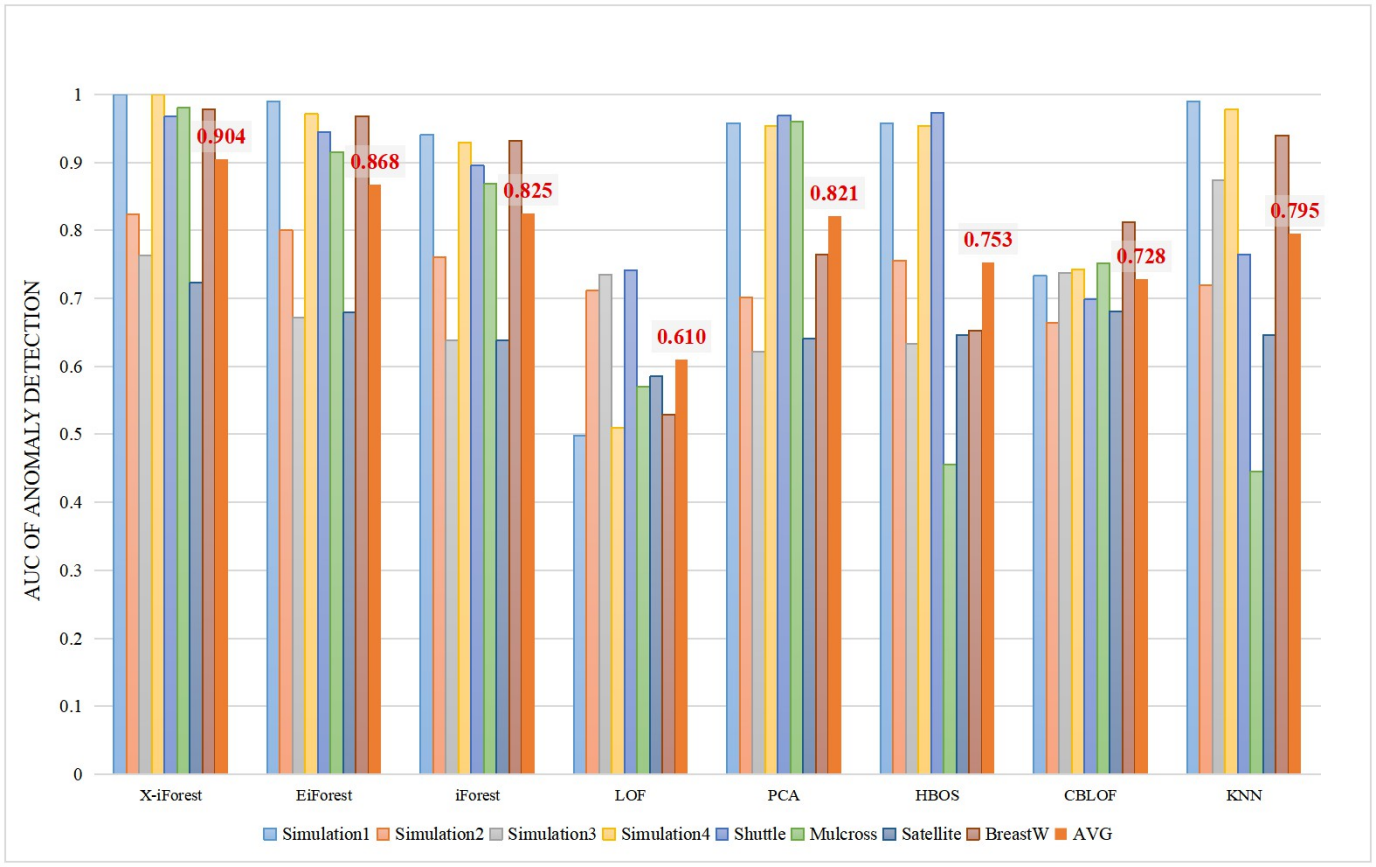
The AUC results of the proposed algorithm X-iForest and others algorithms.

Table 2 counts the *AUC* results of all algorithms. The bold font in the table indicates the best performance in the horizontal experiment. X-iForest performs the best in 6 out of 8 datasets. X-iForest maintains a high AUC in each data set, which also means that the algorithm has high performance and good robustness. Fig 3 shows a histogram of *AUC* results. Based on the average *AUC* value, iForest can maintain high detection accuracy and stability with different datasets. The improvement of X-iForest on the basis of the iForest enables an 8.1% improvement in the average *AUC* in the 8 experimental datasets over that of the iForest, which means that the former has better detection accuracy and better robustness.

**Table 2:** AUC of X-iForest and other algorithms.

Dataset	X-iForest	EiForest	iForest	LOF	PCA	HBOS	CBLOF	KNN
Simulation 1	**1.0**	0.99	0.941	0.498	0.958	0.957	0.733	0.99
Simulation 2	**0.823**	0.8	0.761	0.711	0.701	0.756	0.664	0.719
Simulation 3	0.763	0.672	0.638	0.735	0.621	0.633	0.738	**0.874**
Simulation 4	**1.0**	0.971	0.929	0.51	0.954	0.954	0.743	0.978
Shuttle	0.968	0.945	0.895	0.741	0.969	**0.973**	0.699	0.765
Mulcross	**0.98**	0.915	0.868	0.57	0.96	0.455	0.752	0.445
Satellite	**0.723**	0.679	0.638	0.585	0.641	0.646	0.681	0.646
BreastW	**0.978**	0.968	0.932	0.529	0.764	0.653	0.812	0.94

**Fig 4:**
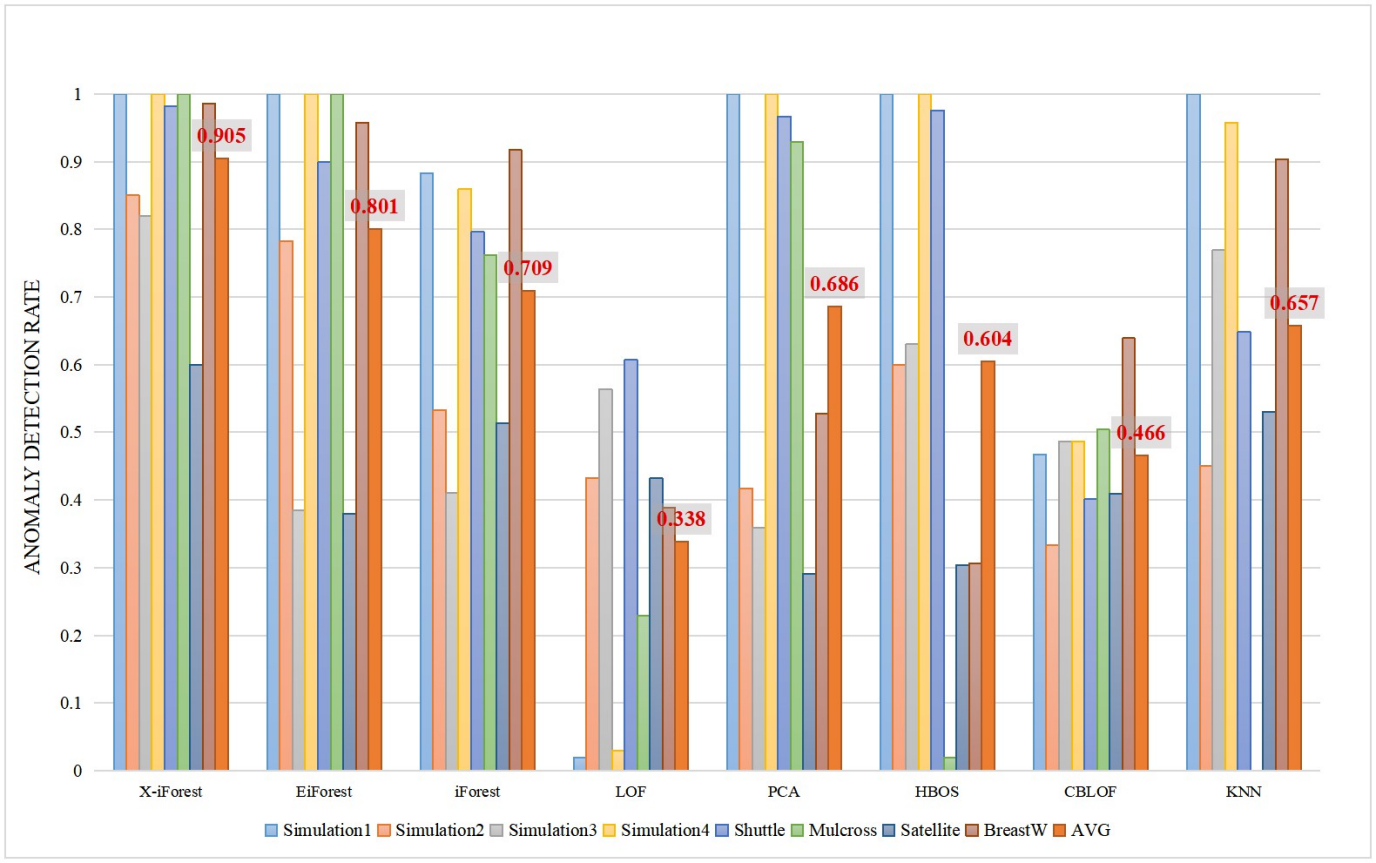
The ADR results of the proposed algorithm X-iForest and others algorithms.

The *ADR* is an important indicator for industrial applications, including network traffic anomaly detection applications. We compared the *ADR*s of X-iForest and other algorithms, and the results are shown in Table 3. X-iForest has the highest *ADR* in all 8 datasets, which shows that X-iForest can complete the anomaly detection task well. Fig 4 shows the histogram of *ADR* results. The average values indicate that the performance of X-iForest is very stable and far better than those of the other algorithms. The results from the *AUC* experiments show that the EiForest algorithm has a good performance and robustness. However, the *ADR* experimental results show that EiForest did not achieve high *ADR* values in the Simulation2 and Satellite Dataset. The average *ADR* of X-iForest is 19.5% higher than that of iForest and 10.1% of EiForest, which means that X-iForest can better complete the anomaly detection task.

**Table 3:** ADR of X-iForest and other algorithms.

Dataset	X-iForest	EiForest	iForest	LOF	PCA	HBOS	CBLOF	KNN
Simulation 1	**1.0**	1.0	0.883	0.02	1.0	1.0	0.467	1.0
Simulation 2	**0.85**	0.783	0.533	0.433	0.417	0.6	0.333	0.45
Simulation 3	**0.82**	0.385	0.41	0.564	0.359	0.63	0.487	0.769
Simulation 4	**1.0**	1.0	0.86	0.03	1.0	1.0	0.487	0.957
Shuttle	**0.982**	0.899	0.796	0.607	0.967	0.976	0.401	0.648
Mulcross	**1.0**	1.0	0.762	0.229	0.929	0.019	0.504	0.0
Satellite	**0.6**	0.38	0.514	0.432	0.291	0.304	0.409	0.53
BreastW	**0.986**	0.958	0.917	0.389	0.528	0.306	0.639	0.903

Based on the results, X-iForest has the best performance in both simulation datasets and the real datasets, maintaining high *AUC* and *ADR* performance, which shows that X-iForest can complete anomaly detection tasks well while maintaining high accuracy. The *AUC* of X-iForest in Simulation3 with a small sample size is not the highest, and there is no large gap with other algorithms. We speculate that X-iForest may not be the best choice for the small sample data set. However, the *ADR* of Simulation 3 shows that X-iForest still accomplishes the anomaly detection task well. Although the Satellite dataset is characterised by high-dimensional and massive volume, the relatively concentrated distribution of the data and the close distribution of outliers and normal values result in many swing points in the data, which means that it is more difficult to do anomaly detection on this dataset. The X-iforest algorithm does not achieve high AUC(0.723) and ADR(0.6) on Satellite dataset, but it still performs much better than other algorithms. Compared to other algorithms, X-iForest shows robustness to different types of data, especially large-scale high-dimensional data, which also proves that X-iForest can be well applied to network health analysis and the field of abnormal network traffic detection.

## Conclusion

The complex network environment and the surge of traffic data make the detection of network traffic anomalies a considerable challenge facing enterprises today. Due to the varying network environment, the definition and distribution of normal and abnormal data have also varied, resulting in a lack of sufficient labelled network traffic data for enterprises. In addition to the explosive growth of traffic data, the current unsupervised anomaly detection algorithms commonly used in industrial applications cannot be well implemented in a real complex network environment. The main research purpose of this paper is to realize the application of unsupervised algorithms in actual dynamic network environments. It is hoped that even when the abnormal ratio of the training data is unknown, the abnormal traffic data in the network traffic can still be found quickly and accurately. In this paper, we combine iForest and X-means to propose the novel algorithm X-iForest. Experiments prove that X-iForest exhibits high accuracy and high performance with massive data in complex networks. The experimental results meet our expectations, and the performance of the X-iForest algorithm is better than that of the iForest algorithm with a precise abnormal ratio. X-iForest has an average AUC score of 8.1% higher than iForest and an average ADR score of 19.5% higher across the 8 datasets. Compared with other algorithms, X-iForest also shows excellent accuracy and robustness without the need to provide precise abnormal ratios. The average AUC score of X-iForest is 15.1% higher than the average score of other algorithms, and the average ADR score of X-iForest reaches 90.4% in the ADR score, while the average ADR score of other algorithms is only 57.6%. This represents a significant advantage of X-iForest and can be well applied to the task of network traffic anomaly detection.

In the next step, we plan to deploy the X-iForest algorithm on a load balancer based on the Flink framework, making full use of the distributed stream processing features of the Flink framework to generate multiple trees in an iForest by placing them on different nodes to speed up training and detection and further validate the performance of the algorithm in real-world network scenarios.
